# On the Role of Calcium-Permeable AMPARs in Long-Term Potentiation and Synaptic Tagging in the Rodent Hippocampus

**DOI:** 10.3389/fnsyn.2019.00004

**Published:** 2019-03-14

**Authors:** Pojeong Park, Heather Kang, Thomas M. Sanderson, Zuner A. Bortolotto, John Georgiou, Min Zhuo, Bong-Kiun Kaang, Graham L. Collingridge

**Affiliations:** ^1^Department of Biological Sciences, College of Natural Sciences, Seoul National University, Seoul, South Korea; ^2^Department of Brain and Cognitive Sciences, College of Natural Sciences, Seoul National University, Seoul, South Korea; ^3^Department of Physiology, Faculty of Medicine, University of Toronto, Toronto, ON, Canada; ^4^Lunenfeld-Tanenbaum Research Institute, Mount Sinai Hospital, Toronto, ON, Canada; ^5^Centre for Synaptic Plasticity, School of Physiology, Pharmacology and Neuroscience, University of Bristol, Bristol, United Kingdom

**Keywords:** NMDA receptor, long-term potentiation, hippocampus, calcium-permeable AMPA receptor, PKA, protein synthesis, synaptic tagging

## Abstract

Classically, long-term potentiation (LTP) at hippocampal CA1 synapses is triggered by the synaptic activation of NMDA receptors (NMDARs). More recently, it has been shown that calcium-permeable (CP)-AMPARs can also trigger synaptic plasticity at these synapses. Specifically, their activation is required for the PKA and protein synthesis dependent component of LTP that is typically induced by delivery of spaced trains of high frequency stimulation. Here we present new data that build upon these ideas, including the requirement for low frequency synaptic activation and NMDAR dependence. We also show that a spaced theta burst stimulation (sTBS) protocol induces a heterosynaptic potentiation of baseline responses via activation of CP-AMPARs. Finally, we present data that implicate CP-AMPARs in synaptic tagging and capture, a fundamental process that is associated with the protein synthesis-dependent component of LTP. We have studied how a sTBS can augment the level of LTP generated by a weak TBS (wTBS), delivered 30 min later to an independent input. We show that inhibition of CP-AMPARs during the sTBS eliminates, and that inhibition of CP-AMPARs during the wTBS reduces, this facilitation of LTP. These data suggest that CP-AMPARs are crucial for the protein synthesis-dependent component of LTP and its heterosynaptic nature.

## Introduction

Long-term potentiation (LTP) has been most extensively studied at the excitatory synapses made between CA3 and CA1 pyramidal neurons (see Bliss et al., 2018 for a recent review). At these synapses three distinct forms of NMDAR-dependent, transcriptionally independent forms of synaptic potentiation have been identified that overlap in time; short-term potentiation (STP), LTP1 and LTP2. The latter form of synaptic plasticity is defined by its dependence on the activation of PKA and protein synthesis (Frey et al., 1993; Matthies and Reymann, 1993; Frey and Morris, 1998; Reymann and Frey, 2007; Bliss et al., 2018). Recently, we have shown that CP-AMPARs are also involved specifically in this form of LTP (Park et al., 2016). These data are summarized in the associated review article (Park et al., 2018). In the present study we have built upon these observations in three ways. We have further investigated the need for synaptic stimulation, delivered after the induction of LTP2, for its expression. In addition, we have evaluated the need to activate NMDARs during the second and third episodes of TBS for the induction of LTP2. We also address the hypothesis that CP-AMPARs participate in the heterosynaptic LTP that accompanies LTP2.

An important concept in synaptic plasticity is the synaptic tag and capture (STC) hypothesis (Frey and Morris, 1997), which was developed to explain how the protein synthesis-dependent component of LTP retained input-specificity given the need for proteins to be delivered to synapses from the soma. An intriguing key observation arising from the initial description of the STC hypothesis is that a weak tetanus capable of inducing a small, protein synthesis-independent LTP (i.e., LTP1) could be converted into a larger protein synthesis-dependent LTP (i.e., LTP2) if a strong tetanus was delivered to an independent input (to induce LTP2 on that input) within a critical time window. In the initial experiments LTP2 was induced by a strong tetanus 60 min before a weak tetanus was delivered to the second input. The mechanistic explanation of this observation is that the strong tetanus engaged the protein synthesis machinery which generated *plasticity related proteins* (PRPs) and the weak tetanus set up a *synaptic tag* that captured some of these PRPs to establish LTP2 at the weak input. Since this pioneering work, there has been considerable effort devoted to identifying the synaptic tag and the PRPs (Frey and Morris, 1998; Barco et al., 2002; Navakkode et al., 2004; Sajikumar and Frey, 2004; Sajikumar et al., 2005, 2007, 2009, 2014; Alarcon et al., 2006; Huang et al., 2006; Young et al., 2006; Ishikawa et al., 2008; Okada et al., 2009; Ramachandran and Frey, 2009; Cai et al., 2010; Redondo et al., 2010; Sajikumar and Korte, 2011; Li et al., 2012; Okuno et al., 2012; Shires et al., 2012; see also Govindarajan et al., 2006; Redondo and Morris, 2011; Rogerson et al., 2014 for reviews). Since we have found that CP-AMPARs are required for LTP2 (Park et al., 2016) we wondered if these receptors are also necessary to initiate the protein synthesis machinery that generates these hypothetical PRPs. In addition, since CP-AMPARs are inserted into synapses during LTP we have speculated whether they may be a component of the synaptic tag machinery (Plant et al., 2006). In the present study we have tested these two hypotheses. We have found that the activation of CP-AMPARs during a spaced theta burst stimulus (TBS) that initiates LTP2 (together with other forms of potentiation) locally, is also required to facilitate LTP on an independent input induced by a weak TBS. In other words, CP-AMPARs are required to initiate PRPs. In addition, we find that CP-AMPARs contribute to the facilitation of LTP at the weak input, indicating that they also serve to “tag” the synapses. Therefore, we can conclude that CP-AMPARs are a fundamental component of the STC hypothesis.

## Materials and Methods

**FIGURE 1:**
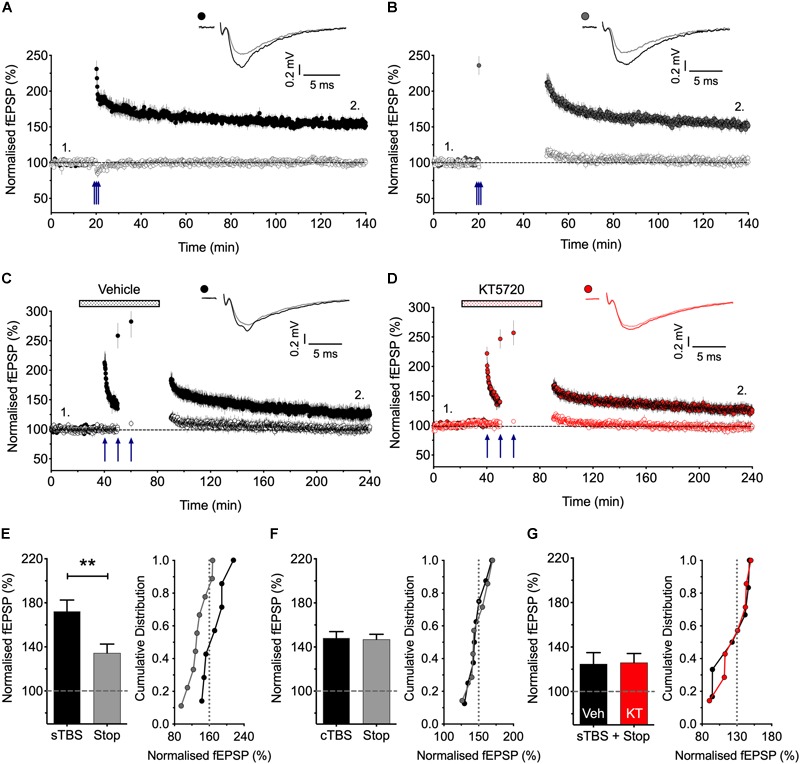
Stimulation post-TBS is not required for LTP1. **(A)** Input specific LTP induced by cTBS from 8 experiments. **(B)** Similar LTP despite a 30 min pause in stimulation (after a test response following TBS to estimate STP; *n* = 7). **(C,D)** Equivalent sTBS experiments except that either vehicle **(C)** or KT5720 (1 μM) **(D)** was applied during the TBS from 6 and 7 animals, respectively. Note that KT has no effect on the residual LTP induced by sTBS when there is a pause in post-TBS stimulation. **(E)** Quantification of sTBS experiments (2 h post TBS). Data replotted from Park et al. (2016). **(F)** Quantification of cTBS experiments (2 h post TBS). **(G)** Quantification of the sTBS with stop stimulation experiments (3 h post TBS) performed either in the presence of KT5720 (KT) or vehicle (Veh). Sample traces are averages of 5 consecutive responses obtained from where indicated by numbers. ^∗∗^*p* < 0.01 vs. control.

**FIGURE 2:**
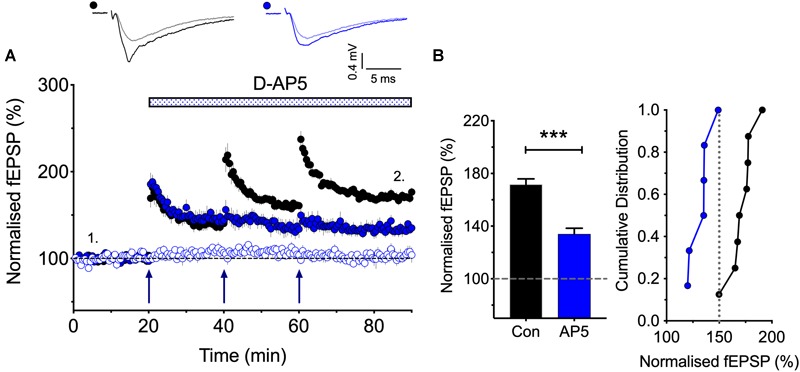
The synaptic activation of NMDARs is required post TBS1 to induce LTP2. **(A,B)** D-AP5 (50 μM) was applied immediately following the first episode of TBS (blue) and completely blocked the induction of additional LTP. Control LTP (black; Con) superimposed for the ease of comparison. The quantification in **(B)** was obtained 30 min post the third TBS (*n* = 8 and 6 for vehicle vs. D-AP5). ^∗∗∗^*p* < 0.001 vs. control.

Experiments were performed as described in Park et al. (2016). Briefly, transverse hippocampal slices (400 μm) were prepared from male Sprague-Dawley rats (experiments reported in Figure 1, 2) or C57BL/6 mice (10–12 weeks of age) using a vibratome (Leica, VT1200S). The CA3 region was cut, with a scalpel blade, to suppress the upstream neuronal excitability, and the slices were transferred to an incubation chamber that contained the recording solution (artificial cerebrospinal fluid, ACSF; mM): 124 NaCl, 3 KCl, 26 NaHCO_3_, 1.25 NaH_2_PO_4_, 2 MgSO_4_, 10 D-glucose and 2 CaCl_2_ (carbonated with 95% O_2_ and 5% CO_2_). Slices were allowed to recover at 32–34°C for 30 min, and then maintained at 26–28°C for a minimum of 1 h before recordings were made.

Hippocampal slices were continuously perfused at 2–4 mL/min with the oxygenated ACSF at 32°C. Two bipolar stimulating electrodes were positioned in stratum radiatum on either side of the recording electrode at approximately the same distance from the cell body layer. Two independent Schaffer collateral-commissural pathways (SCCPs) were stimulated alternately to obtain the evoked synaptic responses, each at a frequency of 0.1 Hz (or 0.033 Hz for data shown in Figure 2). The independence of the two inputs was verified by using paired-pulse analysis. The initial slope of evoked fEPSPs (V/s) was monitored and analyzed using WinLTP (Anderson and Collingridge, 2007). Following a stable baseline period of at least 20 min, LTP was induced using theta-burst stimulation (TBS) delivered at the same basal stimulus intensity and pulse width (0.1 ms; constant voltage stimulator). An episode of TBS comprised 5 bursts at 5 Hz, with each burst composed of 5 pulses at 100 Hz (i.e., 25 pulses in total). For compressed (c) TBS, three TBS episodes were delivered with an inter-episode interval of 10 s. For spaced (s) TBS, the same number of episodes were given but at a 10 or 20 min interval. A weak TBS comprised 3 bursts at 5 Hz (i.e., a total of 15 pulses). Representative sample traces are an average of five consecutive responses, collected from typical experiments (stimulus artifacts were blanked for clarity).

Drugs were prepared as frozen stock solutions (stored below -20°C). Used compounds were: (9R,10S,12S)-2,3,9,10,11,12-hexahydro-10-hydroxy-9-methyl-1-oxo-9,12-epoxy-1H-diindolo[1,2,3-fg:3′,2′,1′-kl]pyrrolo[3,4-i][1,6]benzodiazocine-10-carboxylic acid, hexyl ester (KT5720; Hello Bio); N,N,H,-trimethyl-5-[(tricyclo(3.3.1.13,7)dec-1-ylmethyl)amino]-1-pentanaminiumbromide hydrobromide (IEM-1460; Hello Bio); D-2-amino-5-phosphonopentanoate (D-AP5; Hello Bio); Anisomycin (Hello Bio).

All treatment groups were interleaved with control experiments. Data are presented as mean ± SEM (standard error of the mean). Responses were normalized to the baseline prior to LTP induction. Statistical significance was assessed using (two-tailed) Student’s *t*-test or one-way ANOVA with Bonferroni’s correction as appropriate; the level of significance is denoted as follows: ^∗^*p* < 0.05, ^∗∗^*p* < 0.01, and ^∗∗∗^*p* < 0.001.

## Results

### A Comparison of LTP Induced by Spaced and Compressed Induction Protocols

In previous work we have compared tetanic or TBS induction protocols arranged in either compressed or spaced patterns (Park et al., 2014, 2016). Here we have used three different TBS induction protocols (see Methods for details):

(i) Compressed (c) TBS (3 episodes of theta with an inter-episode interval of 10 s; 75 stimuli in total).
(ii) Spaced (s) TBS (3 episodes of theta with an inter-episode interval of 10–20 min; 75 stimuli in total).
(iii) Weak (w) TBS (1 episode of theta; 15 stimuli in total).

### Differential Effects of a Pause in Stimulation on LTP1 and LTP2

There is controversy as to whether or not low frequency stimulation is required to activate CP-AMPARs during a short period of time following the activation of NMDARs for the generation of LTP (Plant et al., 2006; Adesnik and Nicoll, 2007; Gray et al., 2007), and so we have therefore re-investigated this issue (Figure 1). We reported previously (Park et al., 2016) that if we paused stimulation for 30 min shortly after delivering sTBS then the resultant LTP was smaller than where stimulation was maintained throughout. These previous data are summarized in Figure 1E. We now report the effects of an equivalent 30 min pause in stimulation following a cTBS: as shown in Figure 1A,B,F the level of LTP was indistinguishable with or without a pause in stimulation. We interpret these results as follows: LTP1 (induced by cTBS) does not require stimulation post induction. In contrast, LTP2 does require stimulation post induction, such that when stimulation is paused then only LTP1 is induced. If this residual LTP is indeed LTP1, then it should be insensitive to a PKA inhibitor. Consistent with this idea, we found that a PKA inhibitor, KT5720 (1 μM; KT), had no effect on the residual LTP induced by sTBS followed by a pause in stimulation (Figure 1C,D,G). In conclusion, there exists a critical period following sTBS that requires low frequency synaptic activation and the synaptic expression of CP-AMPARs to generate the PKA-dependent form of LTP (i.e., LTP2). The simplest explanation for the requirement of low frequency stimulation is that this synaptic activation drives Ca^2+^ through CP-AMPARs and that this Ca^2+^ signal is required for the *de novo* protein synthesis.

### The Role of NMDARs in the Induction of LTP2

According to the model that we have proposed to explain the role of CP-AMPARs in LTP, the first TBS induces LTP1 and primes for LTP2, by driving CP-AMPARs into the perisynaptic plasma membrane (see Park et al., 2018). A question left unanswered though is how does the subsequent TBS drive these CP-AMPARs into the synapse. As a first step in addressing this question we asked whether the subsequent TBS episodes are needed to activate NMDARs or not for LTP2 to be induced. In other words, are these subsequent TBS episodes required to activate NMDARs to drive the CP-AMPARs from the perisynaptic to the synaptic membrane? Or do these TBS episodes activate an NMDAR-independent process. For example, they might deliver sufficient L-glutamate to activate the perisynaptically located CP-AMPARs to trigger LTP2. The latter is a possible scenario given that CP-AMPARs can trigger LTP in the presence of NMDAR antagonists under certain conditions (Jia et al., 1996; Whitehead et al., 2013). To distinguish between these two possibilities, we determined whether an NMDAR antagonist blocks the induction of LTP2 when delivered immediately after the first TBS (Figure 2). We found that D-AP5 fully blocked the formation of any additional LTP. These results show, therefore, that the synaptic activation of NMDARs is required for more than just the priming TBS; for example, they may be required to drive the CP-AMPARs from the perisynaptic to the synaptic membrane.

### The Involvement of CP-AMPARs in Heterosynaptic Potentiation

**FIGURE 3:**
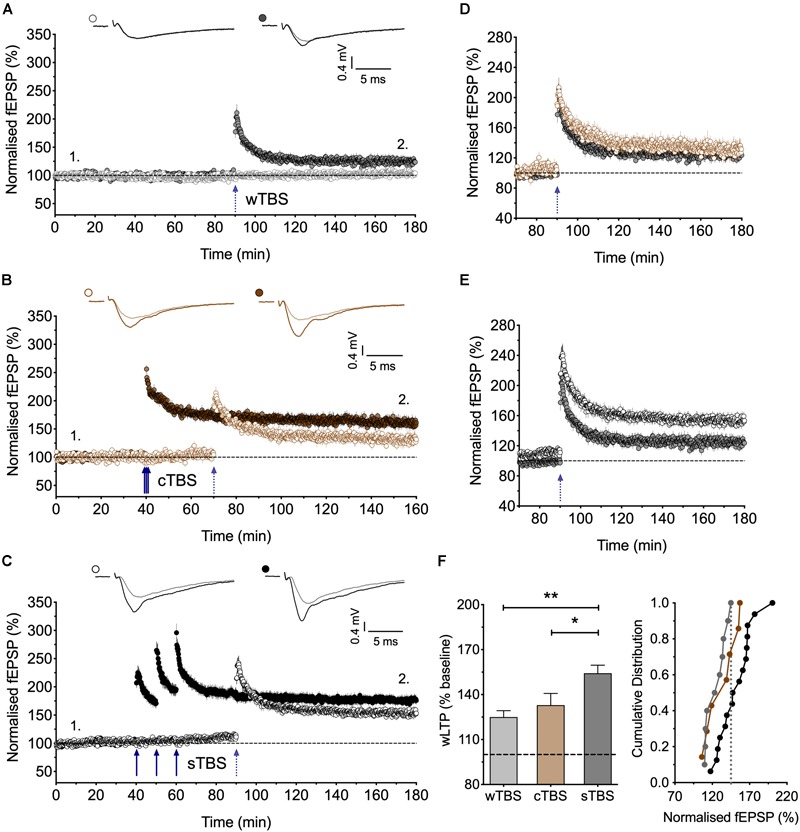
Heterosynaptic facilitation of LTP by delivery of sTBS to an independent input. **(A)** Input-specific LTP induced by a weak TBS (wTBS; 3 bursts of 15 stimuli) from 10 experiments (filled circles); non-tetanized independent input (open circles). **(B)** Similar LTP in the weak input when preceded by a cTBS delivered to an independent input, 30 min previously (*n* = 7). **(C)** Facilitation of LTP induced by the wTBS when preceded by a sTBS delivered to an independent input, 30 min previously (*n* = 16). **(D,E)** Superimposition of the LTP induced by the wTBS (data from **A,B** and **A,C**) to highlight the lack of effect of prior cTBS **(D)** in contrast to the heterosynaptic facilitation of LTP by prior sTBS **(E)**. **(F)** Additional quantification of the data in **(A–C)**. ^∗^*p* < 0.05; ^∗∗^*p* < 0.01; comparisons vs. sTBS.

**FIGURE 4:**
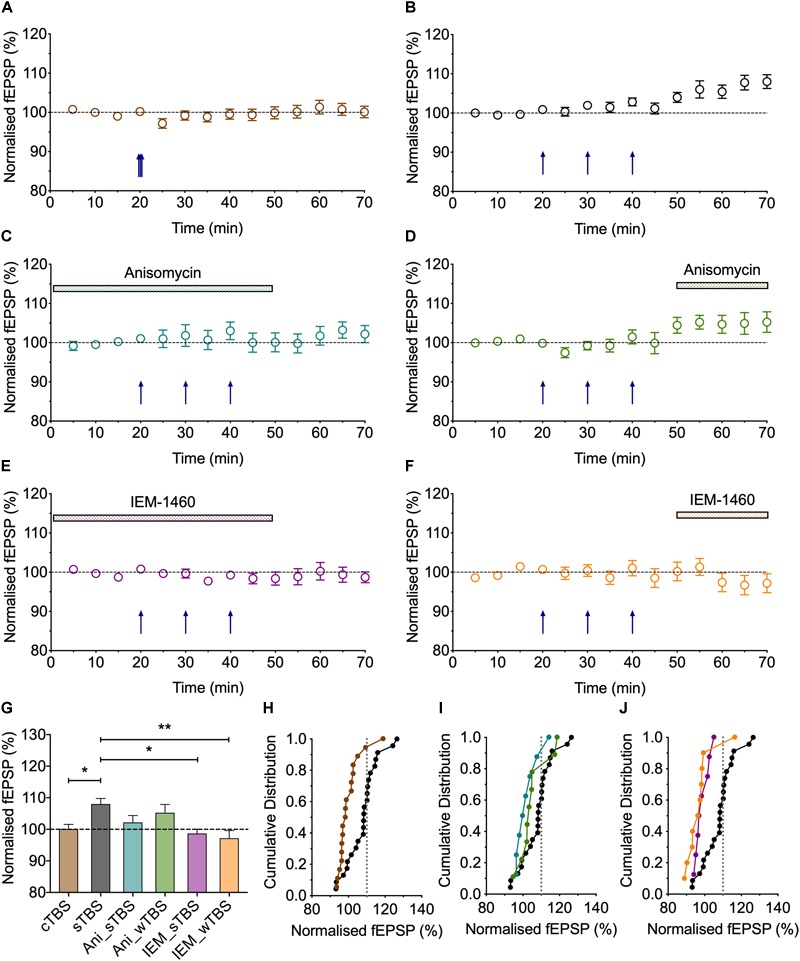
Heterosynaptic LTP induced by a sTBS. **(A,B)** Pooled data of the non-conditioned inputs in response to delivery of **(A)** cTBS (*n* = 18) and **(B)** sTBS (*n* = 23). Data replotted from Figure 1, 3 and Park et al. (2016). **(C–F)** Pooled data of the non-conditioned inputs for sTBS in anisomycin (Ani, 30 μM; *n* = 8), **(D)** anisomycin delivered 10 min after sTBS (*n* = 9), **(E)** sTBS in IEM-1460 (IEM, 30 μM; *n* = 8) and **(F)** IEM delivered 10 min after sTBS (*n* = 10). Data replotted from Figure 5, 6. Each point is the average of responses recorded over a 5 min period. **(G–J)** Summary data with cumulative plots, quantified after 30 min of heterosynaptic LTP induction. ^∗^*p* < 0.05; ^∗∗^*p* < 0.01; comparisons vs. sTBS.

A consistent observation was that whereas LTP induced by a cTBS was invariably entirely input specific (Figure 1A, 3B, 4A), there was a small heterosynaptic potentiation of baseline responses following a sTBS induction protocol delivered to the other input (Figure 3C, 4B). We believe that this represents a genuine, albeit small, heterosynaptic potentiation since before commencing the experiment we invariably confirmed the independence of the two inputs by the lack of heterosynaptic paired-pulse facilitation. In addition, previously published data from our group (Park et al., 2014, 2016) and those of others (e.g., Frey and Morris, 1997; Barco et al., 2002) have also documented a long-lasting heterosynaptic potentiation using spaced, but not compressed, induction protocols. Hence, like LTP2, it is the timing of the bursts that is critical for its generation. Inclusion of the protein synthesis blocker, anisomycin (30 μM), delivered either during or after the sTBS, established that the heterosynaptic LTP requires *de novo* protein synthesis for its induction (Figure 4C, 5A) but not its maintenance (Figure 4D, 5C). In addition, we evaluated the hypothesis that it requires the insertion of CP-AMPARs using the well characterized antagonist IEM-1460 (30 μM; IEM); treatment with IEM prevented the heterosynaptic potentiation when applied during the sTBS (Figure 4E, 6A) or added shortly after the sTBS (Figure 4F, 6C). These data are further quantified in Figure 4G–J. One interpretation of these findings is that the insertion of CP-AMPARs during the sTBS is not input-specific due to spread of Ca^2+^ and cAMP/PKA to neighboring synapses. Another possibility is that the effects of the *de novo* protein synthesis extend beyond the synapses where it is triggered.

**FIGURE 5:**
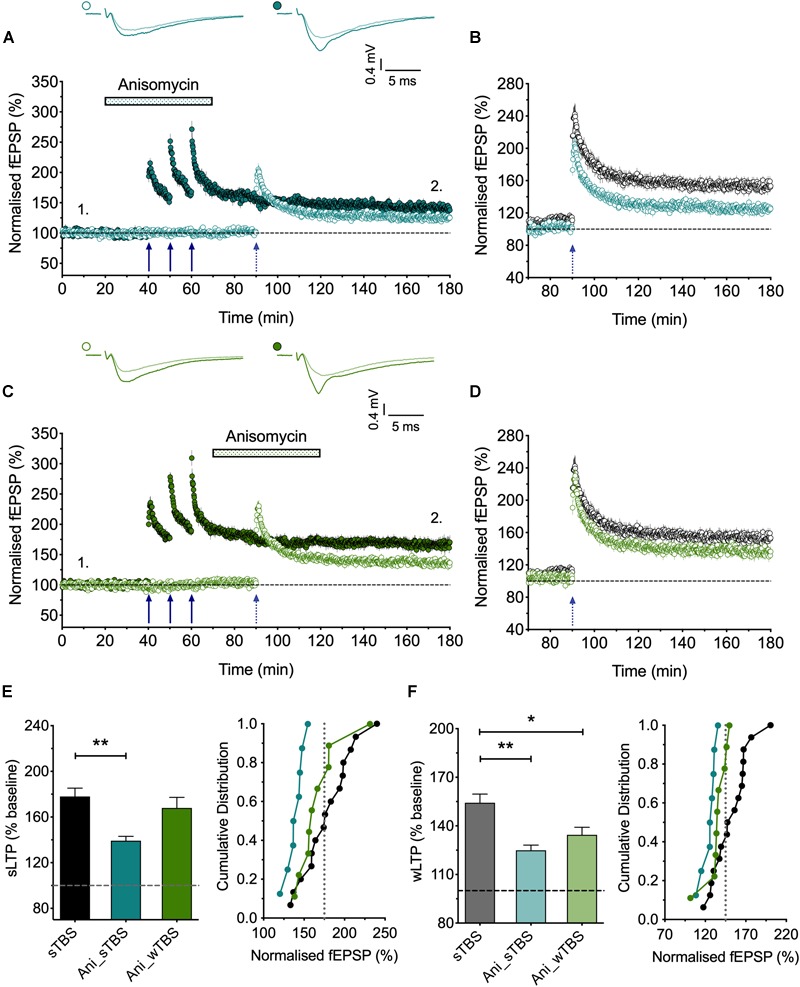
The role of protein synthesis in heterosynaptic facilitation of LTP. **(A)** Anisomycin (30 μM; Ani) applied during the sTBS prevents heterosynaptic facilitation of LTP (*n* = 8). **(B)** Superimposition of the LTP induced by wTBS for anisomycin-treated (blue; from **A**) and untreated controls (black; replotted from Figure 3E). **(C)** Anisomycin applied during the wTBS reduces heterosynaptic facilitation of LTP (*n* = 9). **(D)** Superimposition of the LTP induced by wTBS for anisomycin-treated (green; from **C**) and untreated controls (black; replotted from Figure 3E). **(E)** Quantification (2 h post sTBS) of the effects of anisomycin on the LTP induced by sTBS showing the timing-dependent inhibition of LTP2. **(F)** Quantification (90 min post wTBS) of the effects of anisomycin on the LTP induced by wTBS. ^∗^*p* < 0.05; ^∗∗^*p* < 0.01; comparisons vs. sTBS.

**FIGURE 6:**
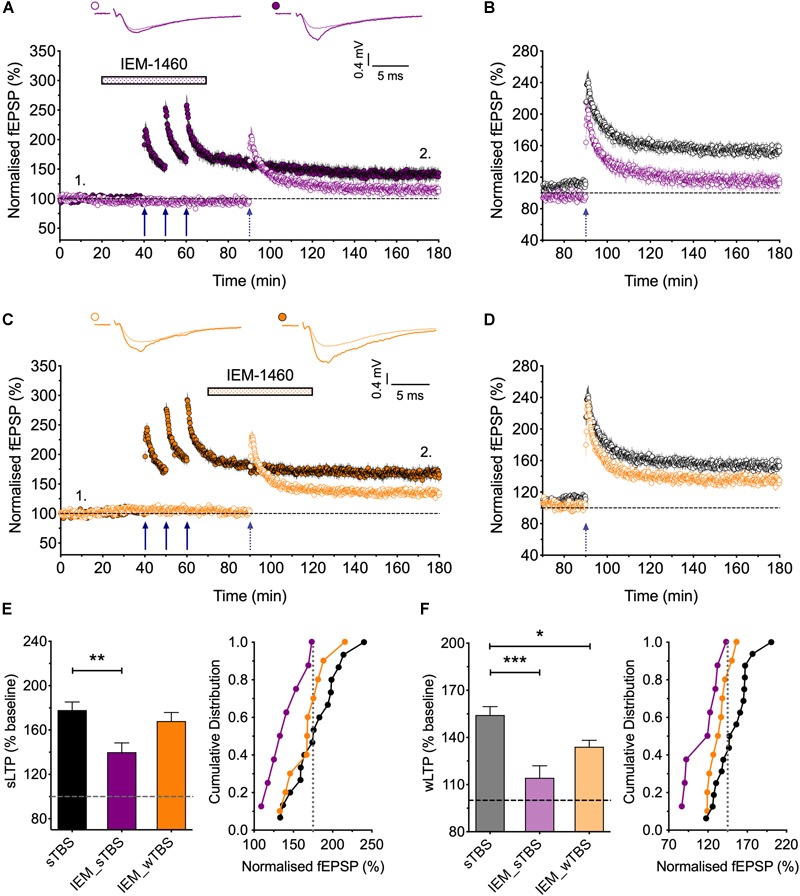
Evidence that synaptic tagging and capture involves CP-AMPARs. **(A)** IEM-1460 (30 μM; IEM) applied during the sTBS prevents synaptic tagging (*n* = 8). **(B)** Superimposition of the LTP induced by wTBS to illustrate the magnitude of the effect of IEM treatment (purple; from **A**) compared to untreated controls (black; replotted from Figure 3E). **(C)** IEM applied during the wTBS reduces synaptic tagging (*n* = 10). **(D)** Superimposition of the LTP induced by wTBS to illustrate the magnitude of the effect of IEM treatment (orange; from **C**) compared to untreated conditions (black; replotted from Figure 3E). **(E)** Quantification (2 h post sTBS) of the effects of IEM on the LTP induced by sTBS, showing its timing-dependent inhibition of LTP2. **(F)** Quantification (90 min post wTBS) of the effects of IEM on the LTP induced by wTBS. ^∗^*p* < 0.05; ^∗∗^*p* < 0.01; ^∗∗∗^*p* < 0.001; comparisons vs. sTBS.

### sTBS Triggers Heterosynaptic Facilitation of LTP

Since sTBS, but not cTBS, triggers LTP2 by engaging *de novo* protein synthesis and since this effect is not entirely input-specific, we hypothesized that sTBS may also initiate the STC process to augment the induction of LTP in an independent input triggered by a wTBS. To investigate this possibility, we either delivered a weak TBS (wTBS; 15 stimuli) alone, which generated modest LTP (125 ± 4% of baseline; *n* = 10; Figure 3A,F), or we preceded the wTBS with a sTBS (75 stimuli) delivered 30 min previously to an independent input. The latter resulted in a much greater LTP in response to the wTBS (154 ± 5% of baseline; *n* = 16, ^∗∗^*p* = 0.002, one-way ANOVA, *F*_(2,30)_ = 8.01; Figure 3C,E,F). As a control experiment, we also delivered a cTBS (75 stimuli) 30 min before the wTBS but this did not lead to a facilitated LTP (133 ± 8% of baseline; *n* = 7, *p* = 0.79, one-way ANOVA; Figure 3B,D,F). These observations are in agreement with the STC hypothesis of Frey and Morris (1997). They show further that the timing of the initial “priming” stimuli rather than its strength *per se* is the critical factor.

### The Role of *de novo* Protein Synthesis in Heterosynaptic Facilitation of LTP

The findings that priming is induced by a sTBS, but not a cTBS, is consistent with the notion that the STC process involves *de novo* protein synthesis. To test this directly, we studied the effects of anisomycin delivered during the sTBS (Figure 5). As expected this treatment completely prevented the priming effect (125 ± 3% of baseline; *n* = 8, ^∗∗^*p* = 0.0012, one-way ANOVA, *F*_(2,30)_ = 9.06; Figure 5A,B,F); in interleaved experiments we also applied anisomycin during the wTBS, which significantly reduced, but did not eliminate, the priming effect (135 ± 5% of baseline; *n* = 9, ^∗^*p* = 0.03, one-way ANOVA; Figure 5C,D,F). These data are consistent with the possibility that CP-AMPARs trigger protein synthesis during the sTBS, which primes LTP in response to a wTBS at an independent input. They suggest further that protein synthesis is engaged by the wTBS and is required for the full priming effect.

### The Involvement of CP-AMPARs in “Synaptic Tag and Capture”

CP-AMPARs endow synapses with properties that suggest that they could be part of the STC process. They may be involved in the generation of PRPs and/or they may serve as synaptic tags (Plant et al., 2006). To test the first possibility, we delivered the sTBS in the presence of IEM (Figure 6A,B,F). This treatment completely prevented the heterosynaptic facilitation of LTP (114 ± 8% of baseline; *n* = 8, ^∗∗∗^*p* < 0.001, one-way ANOVA, *F*_(2,31)_ = 11.93), suggesting that the “priming” effect requires the activation of CP-AMPARs.

To test the second possibility, in interleaved experiments, we applied IEM starting 10 min after the delivery of sTBS, at a time when the transiently recruited CP-AMPARs should no longer contribute appreciably to the expression of homosynaptic LTP (Plant et al., 2006); accordingly, IEM had no significant effect on the level of LTP induced by the sTBS, compared to interleaved control experiments. After IEM had been applied for 20 min, to reach a steady-state concentration, we delivered wTBS to the second input (Figure 6C,D,F). In these conditions, the wTBS led to a reduced LTP compared to an untreated, primed input (134 ± 4% of baseline; *n* = 10, vs. 154 ± 5% of baseline; *n* = 16, ^∗^*p* = 0.04, one-way ANOVA).

These data demonstrate that CP-AMPARs are (i) required during the delivery of the sTBS for the priming of the wTBS and (ii) contribute to the facilitation during the wTBS. In other words, CP-AMPARs are required to generate PRPs. Additionally, they may have a role in the induction of LTP at the primed input, though this is not essential. Indeed, one of their functions may be to “tag” heterosynaptic inputs for the generation of LTP2 in response to a weak input within a critical time window, governed by their presence on the plasma membrane.

## Discussion

In this study we have described several new features concerning the role of CP-AMPARs in LTP2, the component of LTP that involves PKA and *de novo* protein synthesis (see Bliss et al., 2018). Firstly, we showed that the requirement for low frequency stimulation following the induction of LTP is specific for LTP2. Next, we demonstrated that during a sTBS protocol the NMDARs need to be activated not just for the first priming TBS but for the subsequent TBS episodes as well. Thirdly, we found that LTP2 is not entirely input-specific but rather there is a small heterosynaptic LTP that involves CP-AMPARs and *de novo* protein synthesis. Finally, we observed that CP-AMPARs influence synaptic plasticity beyond the activated input, implicating them in the STC process. We found that a sTBS, but not a cTBS, initiates a CP-AMPAR- and protein synthesis-dependent process that can enhance LTP in an independent input. These new observations are discussed in turn.

### The Requirement for Low Frequency Stimulation Post TBS for the Induction of LTP

There has been a controversy as to whether stopping stimulation for a brief period following the activation of NMDARs impairs the expression of LTP (Plant et al., 2006) or not (Adesnik and Nicoll, 2007; Gray et al., 2007). Our present findings when considered in conjunction with our previous work (Park et al., 2016; Park et al., 2018) resolves this controversy. We show that LTP2 is sensitive to the pause in stimulation whereas LTP1, induced either using a cTBS or using a sTBS in the presence of a PKA inhibitor, is not. These results are consistent with the idea that once inserted into the synaptic membrane, CP-AMPARs need to be activated by low frequency stimulation to trigger LTP2. The most likely explanation is that Ca^2+^ entry via CP-AMPARs (Morita et al., 2013) is required to trigger protein synthesis.

### The Role of NMDARs for the Induction of LTP

It is well known that the activation of NMDARs is required to trigger LTP at CA1 synapses under most experimental conditions. However, their precise role during spaced induction protocols has not been established. According to our model (Park et al., 2018), the first TBS activates NMDARs and this triggers CaMKII which drives more calcium impermeable-AMPARs into the synapse to induce LTP1. It also activates PKA, presumably via activation of Ca^2+^-sensitive adenylyl cyclase and the formation of cAMP, which results in the insertion of CP-AMPARs at perisynaptic sites to prime for LTP2. The second and third TBS then drive these CP-AMPARs into the synapse where they can be activated by low frequency synaptic transmission to trigger protein synthesis. We asked whether the activation of NMDARs during the second and third TBS is also required for this step in the induction process or not. Since CP-AMPARs can express LTP in the presence of an NMDAR antagonist under certain experimental conditions (Jia et al., 1996; Whitehead et al., 2013), it seemed feasible that this latter step could occur without the activation of NMDARs. However, our new findings show that NMDARs indeed are required for this step of the induction process too. We can conclude, therefore, that the second and third episodes of TBS activate NMDARs to drive CP-AMPARs from perisynaptic to synaptic sites. Whether this is due to activation of CaMKII and/or other Ca^2+^-sensitive steps remains to be established.

### The Role of CP-AMPARs in Heterosynaptic LTP

Although NMDAR-dependent LTP is usually considered to be an input-specific process, there are examples of heterosynaptic alterations. For example, in the first demonstration of synaptic tagging and capture, heterosynaptic potentiation was evident (Frey and Morris, 1997). In the present study we observed a small (∼10%), but consistent, heterosynaptic LTP. This effect was not observed when we delivered cTBS, despite delivering the same number of stimuli (75 in both cases) and inducing LTP of a similar magnitude. Similar to LTP2 at the homosynaptic input, heterosynaptic LTP required activation of CP-AMPARs and *de novo* protein synthesis for its induction and it also appeared to involve the insertion of CP-AMPARs for its expression. The heterosynaptic LTP developed gradually, over a period of 10–20 min. This contrasts with the synaptic potentiation on the homosynaptic input, which is observed immediately and declines rather than grows. But this can be explained by the co-existence of both STP and LTP1 with LTP2 specifically at the inputs receiving the sTBS.

What dictates the extent of heterosynaptic LTP is unknown, but potentially it could be determined by how local NMDAR activation can trigger the necessary Ca^2+^-sensitive processes, such as activation of CaMKII, at remote synapses. In this context, it is worth noting that the synaptic activation of NMDARs can trigger Ca^2+^ release from intracellular stores (Alford et al., 1993; Emptage et al., 1999) and this process could, in principle, extend the influence of the NMDAR-triggered Ca^2+^ signal. Another possibility may be the extent to which *de novo* protein synthesis triggered at one synapse can influence its neighbors. What the function of heterosynaptic LTP may be is also a matter for speculation. One possibility is to create a zone of synapses, presumably surrounding homosynaptically potentiated synapses that are more susceptible to future potentiation by virtue of their increased strength and heightened ability to trigger additional protein synthesis.

### The Role of CP-AMPARs in the STC Hypothesis

Our new observations present evidence that CP-AMPARs are part of the process that generates the PRPs. Although we cannot discount a role for somatic protein synthesis it is likely that, over the time-course of our experiments, CP-AMPARs are triggering protein synthesis from pre-existing mRNAs present at ribosomes in the proximity of the activated synapses. How CP-AMPARs initiate protein synthesis is unknown. However, it is pertinent to note that in GluA2 KO mice, CP-AMPARs trigger an NMDAR-independent LTP via activation of PI3K and ERK (Asrar et al., 2009). Since *de novo* protein synthesis during LTP involves activation of these kinases (Kelleher et al., 2004a,b), in addition to PKA (Huang and Kandel, 1994), it is conceivable that CP-AMPARs can activate these processes in a manner that NMDAR activation alone cannot. This may relate to differences in the kinetics of the associated Ca^2+^ signals (brief, large increases during TBS vs. prolonged smaller increase during low frequency stimulation for NMDARs and CP-AMPARs, respectively) and/or because these receptors elevate Ca^2+^ in different microdomains within synapses.

**FIGURE 7:**
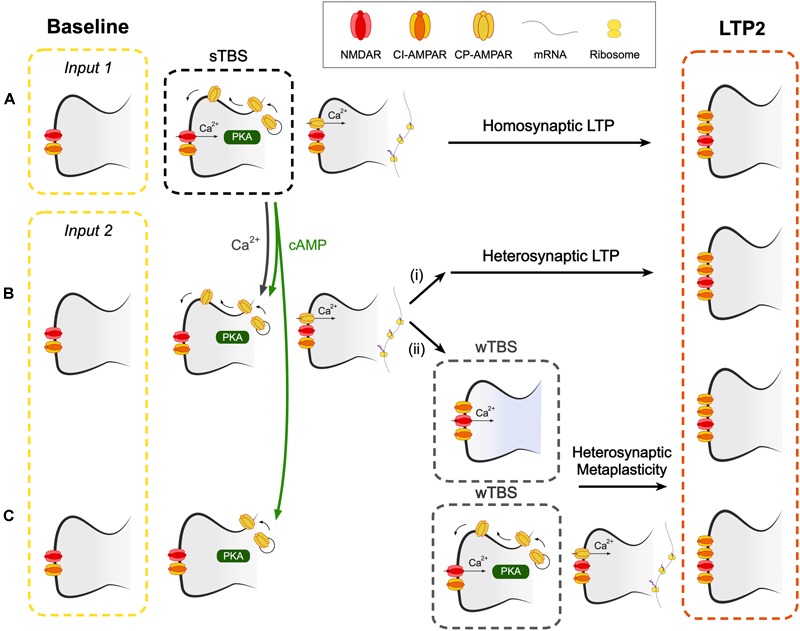
A hypothetical scheme to explain how CP-AMPARs may contribute to heterosynaptic LTP and heterosynaptic metaplasticity. **(A)** A spaced theta burst (sTBS) induction protocol (to input 1), as described in Park et al. (2018), induces LTP2 on the homosynaptic input (LTP1 will also be induced, not shown). **(B)** Spread of Ca^2+^ and cAMP (or PKA), generated during the sTBS, to proximal independent inputs triggers local *de novo* protein synthesis. This leads to (i) heterosynaptic LTP2 and (ii) heterosynaptic metaplasticity. Since protein synthesis has already been triggered, this component of metaplasticity is not affected by inhibitors of CP-AMPARs or protein synthesis. **(C)** At more distal synapses, the spread of cAMP (or PKA), but not Ca^2+^, leads to the insertion of CP-AMPARs into the perisynaptic membrane. wTBS can drive these CP-AMPARs into the synapse and trigger heterosynaptic metaplasticity, via the triggering of local *de novo* protein synthesis. As such, this component of metaplasticity is sensitive to inhibitors of CP-AMPARs and protein synthesis.

The next issue is how CP-AMPARs trigger protein synthesis at independent inputs. One possibility is that the dendritic protein synthesis machinery is, when activated at one group of synapses, engaged over a wider region of the dendritic tree (Govindarajan et al., 2011). This effect might relate to the spread and/or activation of various signals including PKA, Ras-ERK, Rac1 and RhoA activity, which have been shown to occur and spread following the induction of synaptic plasticity (Wu et al., 2001; Hedrick et al., 2016; Tang and Yasuda, 2017). Since PKA is commonly tethered to scaffolding molecules within the proximity of synapses, the spread of its activity may be mediated, or perhaps extended, by cAMP (Bacskai et al., 1993). A model that illustrates how CP-AMPARs may trigger the formation of PRPs at heterosynaptic inputs appears in Figure 7.

Our experiments are also suggestive of an additional role of CP-AMPARs in the STC process since IEM reduced, but did not block, the level of LTP when it was delivered during the wTBS (Figure 6). As noted before (Barco et al., 2002), anisomycin also reduced, but did not block, the heterosynaptic facilitation of LTP when delivered during the wTBS. Therefore it must be concluded that heterosynaptic metaplasticity has two components, one that is sensitive and one that is insensitive to both inhibitors of CP-AMPARs and protein synthesis, when applied during the wTBS. With respect to the insensitive component, we propose that this occurs at proximal inputs where the activation of CP-AMPARs and *de novo* protein synthesis have already been triggered by the sTBS. The wTBS is then required to trigger downstream components of the induction process. In contrast, there may be more distal synapses where CP-AMPARs are trafficked to perisynaptic sites but do not get inserted into the synapse to trigger protein synthesis. The wTBS is then required additionally to drive the synaptic insertion of CP-AMPARs and these then trigger the local *de novo* protein synthesis. Although still a matter for speculation, it is possible that the determinant of whether the heterosynaptic metaplasticity requires activation of CP-AMPARs or not is the extent of the spread of Ca^2+^. At proximal inputs, Ca^2+^ may spread to activate the Ca^2+^ -sensitive steps, such as activation of CaMKII, to drive CP-AMPARs into the synapse. Whereas at distal synapses the Ca^2+^ may not reach this far and so the wTBS is required to deliver the necessary Ca^2+^. In this latter scenario, CP-AMPARs act as a synaptic tag, where they mark surrounding synapses for future LTP2 (Figure 7).

It is relevant to note that the insertion of CP-AMPARs at perisynaptic sites of heterosynaptic inputs could occur if cAMP spreads beyond activated synapses via the PKA-dependent phosphorylation of Ser845 of GluA1. Therefore the two forms of heterosynaptic metaplasticity are governed by the availability of Ca^2+^ (insensitive component) and cAMP (both components). The scheme is consistent with the observation that heterosynaptic facilitation of LTP also requires the activation of PKA during the weak induction protocol (Young et al., 2006). It is also consistent with the tightly regulated, rapid local activation and suppression of translation (for review see Holt and Schuman, 2013) a process that may also involve NMDAR-triggered, Argonaute-mediated regulation of miRNAs (Rajgor et al., 2018).

## Conclusion

The mechanism that we have described here can, perhaps, best be thought of as a form of heterosynaptic metaplasticity. This effect bears some similarities to a homosynaptic form of metaplasticity that is triggered by the synaptic activation of mGluRs (Bortolotto et al., 1994) and also involves protein synthesis (Raymond et al., 2000). Therefore, potentially mGluRs enable homosynaptic metaplasticity while CP-AMPARs confer heterosynaptic metaplasticity on NMDAR-dependent LTP. In summary, CP-AMPARs can be thought of as a trigger to induce PRPs, to potentially comprise one of the PRPs and to constitute the synaptic tag.

## Ethics Statement

The study was carried out in accordance with the recommendations of the local animal care committees under approved animal use protocols in Bristol, Seoul and Toronto (TCP), and in accordance with the respective animal councils/legislation (United Kingdom Home Office/ASPA; Canadian Council on Animal Care/CCAC).

## Author Contributions

PP and HK performed the experiments, analysis and co-wrote and prepared the manuscript. TS, ZB, JG, MZ, and B-KK contributed to the manuscript. GC designed the studies and co-wrote the manuscript.

## Conflict of Interest Statement

The authors declare that the research was conducted in the absence of any commercial or financial relationships that could be construed as a potential conflict of interest.
